# Pathway-Activity Likelihood Analysis and Metabolite Annotation for Untargeted Metabolomics Using Probabilistic Modeling

**DOI:** 10.3390/metabo10050183

**Published:** 2020-05-03

**Authors:** Ramtin Hosseini, Neda Hassanpour, Li-Ping Liu, Soha Hassoun

**Affiliations:** Department of Computer Science, Tufts University, Medford, MA 02155, USA; ramtin.hosseini@tufts.edu (R.H.); Neda.hassanpour@tufts.edu (N.H.); Liping.Liu@tufts.edu (L.-P.L.)

**Keywords:** machine learning, inference, untargeted metabolomics, biological network, metabolic model

## Abstract

**Motivation**: Untargeted metabolomics comprehensively characterizes small molecules and elucidates activities of biochemical pathways within a biological sample. Despite computational advances, interpreting collected measurements and determining their biological role remains a challenge. **Results**: To interpret measurements, we present an inference-based approach, termed Probabilistic modeling for Untargeted Metabolomics Analysis (PUMA). Our approach captures metabolomics measurements and the biological network for the biological sample under study in a generative model and uses stochastic sampling to compute posterior probability distributions. PUMA predicts the likelihood of pathways being active, and then derives probabilistic annotations, which assign chemical identities to measurements. Unlike prior pathway analysis tools that analyze differentially active pathways, PUMA defines a pathway as *active* if the likelihood that the path generated the observed measurements is above a particular (user-defined) threshold. Due to the lack of “ground truth” metabolomics datasets, where all measurements are annotated and pathway activities are known, PUMA is validated on synthetic datasets that are designed to mimic cellular processes. PUMA, on average, outperforms pathway enrichment analysis by 8%. PUMA is applied to two case studies. PUMA suggests many biological meaningful pathways as active. Annotation results were in agreement to those obtained using other tools that utilize additional information in the form of spectral signatures. Importantly, PUMA annotates many measurements, suggesting 23 chemical identities for metabolites that were previously only identified as isomers, and a significant number of additional putative annotations over spectral database lookups. For an experimentally validated 50-compound dataset, annotations using PUMA yielded 0.833 precision and 0.676 recall.

## 1. Introduction

Analyzing cellular responses to perturbations such as drug treatments and genetic modifications promises to elucidate cellular metabolism, leading to improved outcomes in personalized medicine and synthetic biology. Metabolomics has emerged as the new ‘omics’, providing a readout of cellular activity that is most predictive of phenotype. Metabolomics, so far, has played a critical role in advancing applications spanning biomarker discovery [1], drug discovery and development [2], plant biology [3], nutrition [4], and environmental health [5]. Importantly, the advent of *untargeted metabolomics* to measure molecular masses and spectral signatures of thousands of small molecule metabolites for a biological sample allows unprecedented opportunities to characterize the phenotype.

The success of untargeted metabolomics in providing insight into cellular behavior, however, hinges on solving two problems. *Metabolite annotation* concerns associating measured masses with their chemical identities. This problem is challenging, as a particular mass may be associated with multiple chemical formulas (e.g., there are 21,988 known molecules associated with C_20_H_24_N_2_O_3_). There are several techniques for annotating measurements. Database lookups rely on comparing the measured spectral signature against experimentally-generated fragmentation patterns cataloged in reference spectral databases (e.g., METLIN [6], HMDB [7], MassBank [8], NIST [9]). Database coverage, however, is limited as catalogued spectral signatures are obtained experimentally. Alternatively, computational methods that either mimic the ionization and fragmentation process or utilize machine learning techniques (e.g., MetFrag [10], Fragment Identificator (FiD) [11], CFM-ID [12], CSI:FingerID [13]) score the measured spectra against the predicted spectra of molecules in a candidate set. The chemical identity associated with the highest scoring signature(s) is then assigned to the measured spectra. Other annotation techniques exploit the biological context of the measurements. For example, iMet [14] and BioCAn [15] exploit data about local neighborhoods within the network graphs to improve annotation, while EMMF uses the biological context to engineer a candidate set based on enzyme promiscuity [16].

The second problem, *pathway enrichment analysis*, concerns interpreting measurements within their biological context to study coordinated changes arising in response to cellular perturbations. Overrepresentation Analysis (ORA) tools (e.g., MESA [17], MetaboAnalyst [18], MPEA [19]) employ statistical testing (e.g., Fisher’s exact test) to determine if a pathway is enriched in measured metabolites to a degree different than expected by chance when compared to other cellular pathways or those in a reference sample [20]. Pathway enrichment techniques can be broadly classified in two categories. Topological analysis (TA) computes the observed metabolites’ centrality and connectivity, metrics that reflect the importance of a metabolite in the turnover of molecules through a pathway or network (e.g., MetaboAnalyst [18] and IMPaLA [21]). Metabolite annotation and pathway enrichment have traditionally been solved as two independent problems, where pathway enrichment assumes that the chemical identity of each measured mass is known a priori. In general, pathway analysis techniques, therefore, do not adequately address issues related to uncertainty in metabolite annotation. One exception is Mummichog, a set of statistical algorithms that predict functional activity directly from measurements considered significant when compared to those in a reference sample [22].

We present a novel inference-based probabilistic approach, Probabilistic modeling for Untargeted Metabolomics Analysis (PUMA), for interpreting metabolomics measurements. One input to PUMA is the set of measurements that are already processed through metabolomics, e.g., MZmine [23], XCMS [24], CAMERA [25]. Another input is a set of pathways, each consisting of enzymatic reactions and their metabolic products, that are specific to the sample under study. Such pathways can be readily assembled from databases such as KEGG or MetaCyc or others. Using these data, PUMA first calculates the likelihood of activity of metabolic pathways within a biological sample. PUMA then utilizes these predictions to derive probabilistic assignment of measurements to candidate chemical identities. PUMA utilizes inference and approximates posteriors using Gibbs sampling, a Markov Chain Monte Carlo (MCMC) sampling technique [26]. Although inference is a well-known machine learning technique, there were several challenges in developing PUMA including: (1) identifying a suitable generative model that represents the underlying biological process, (2) expressing complex relationships using probability distributions, (3) speeding the inference procedure with complex mathematical marginalization and vectorization, (4) identifying best model parameters, and (5) validating model against the ground truth. Herein, we describe how PUMA addresses such challenges. PUMA is then applied to two data sets collected for Chinese Hamster Ovary (CHO) cells [15] and human urinary samples [27]. Predicted pathway activities are analyzed for biological significance and compared against activity predictions obtained through statistical pathway enrichment analysis. We compare PUMA annotations against those already established by prior analysis of these datasets. For the CHO cell test case, metabolite annotations obtained using PUMA are compared to those published prior [15], including annotations using METLIN [6], HMDB [7], and BioCAn [15]. For the human urinary samples, PUMA annotations are compared to published annotations [27] obtained using spectral databases and experimental validation.

## 2. Methods

### 2.1. Motivating Example

**Figure 1:**
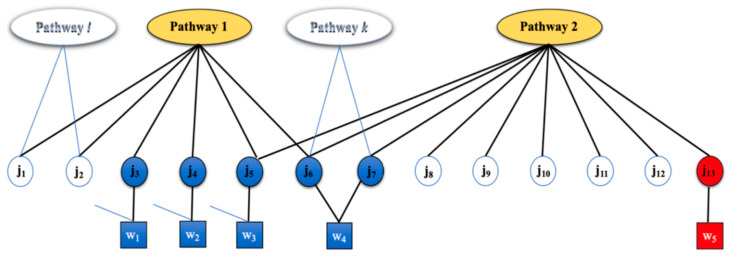
Illustrative example of uncertainty when mapping measurements to metabolites and pathways. Pathways (ovals) are associated with metabolites (circles), which in turn are associated with measurements (square). White circles represent non-measured metabolite with membership in one or more pathways. Blue circles represent measured metabolites that have multiple-pathway memberships (multiple-pathway membership is assumed but not shown for j_3_ and j_4_). The red circle represents a metabolite that has membership in only one pathway. Measurement w_5_ uniquely maps to j_13_, which uniquely maps to Pathway 2, while all other measurements map to multiple metabolites, as shown by solid or dotted lines.

A small example is provided to illustrate challenges in mapping measurements to metabolites and pathways, and to show inference’s ability to address these issues. Figure 1 presents a snippet of a network that shows two pathways (ovals), Pathway 1 and Pathway 2. Metabolites with known chemical identities associated (circles) are either associated with one pathway (red circle) or more than one pathway (blue circles). Measurements (squares) correspond to masses that can be associated with one particular metabolite (red square) or multiple metabolites (blue squares). Not all metabolites within a sample are measured due to either instrument limitations or because they are simply not present in the sample due to biological or environmental factors. Some metabolites are thus not associated with any measurements (white circles), and maybe associated with one or more pathways.

There are two types of uncertainties in interpreting measurements from untargeted metabolomics. One type of uncertainty relates to assignment of metabolites to pathways (circles to ovals, Figure 1). For example, measurement w_3_ is assigned to metabolite j_5_. Because j_5_ is a metabolite common to both Pathways 1 and 2, there is an uncertainty in assignment of the metabolite to the pathways: j_5_ can be the product of activity in either Pathway 1, Pathway 2, or both. The other uncertainty relates to assignment of masses to metabolites, when a mass can map to multiple metabolites (squares to circles, Figure 1). Measurement w_4_ can be attributed to one or two metabolites, j_6_ and j_7_, both sharing the same mass. The uncertainty in assigning w_4_ to metabolites j_6_ and j_7_ manifests in further uncertainty. If w_4_ is associated with j_6_, then it contributes to the activity of Pathway 1 (and/or other pathways with which j_6_ is associated), while, if w_4_ is associated with j_7_, then it clearly ought to contribute to the activity of Pathways 2 (and/or other pathways with which j_7_ is associated). Not all measurements contribute to these uncertainties. For example, measurement w_5_ is unique to metabolite j_13_. In turn, j_13_ is unique to Pathway 2. Some measurements (such as w_5_) clearly contribute more significantly than others (such as w_3_ and w_4_) in determining pathway activities.

Computing pathway activities using an enrichment ratio can be misleading, because it does not take into account the uncertainty in attributing measurements to metabolites and pathways. The enrichment ratio for Pathway 1 can be computed as the ratio of four putatively-measured metabolites divided by six total metabolites in the pathway. While this enrichment ratio seems high, there is little confidence that Pathway 1 is active since all measured metabolites form this pathway could be due to active pathways other than Pathway 1. Pathway 2 has an enrichment ratio equal to 3 divided by 8. The significance or importance of this ratio is unclear. Inference will conclude that Pathway 2 is active with high probability, as it includes a measured metabolite that cannot be attributed to the activity of any other pathway. In contrast to enrichment methods, our inference-based technique considers uncertainties in measurement-metabolite and metabolite-pathway relationships when computing the likelihood of pathway activities. A pathway is considered active, if the likelihood that it generated the observed measurements is above a particular threshold. When we analyze our test cases, we will assume a threshold of 0.5. A user of PUMA may decide to use this threshold or select a more suitable threshold above which pathways are deemed active.

### 2.2. Generative Model

**Figure 2:**
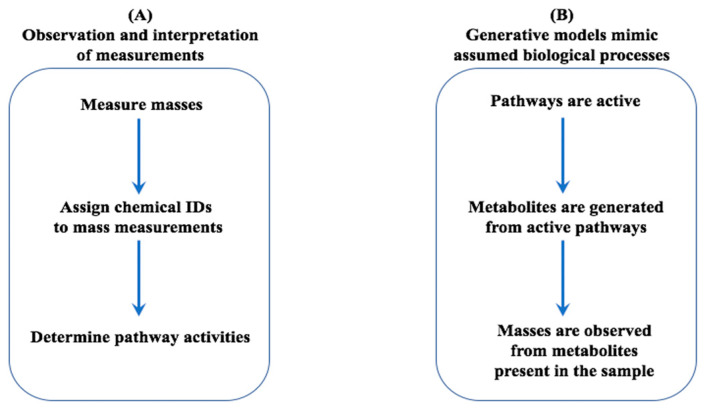
Comparison of a workflow to collect and interpret observations (**A**), and a generative model that captures a biological process (**B**).

To determine pathway activities, an untargeted metabolomics workflow (Figure 2A) begins with collecting measurements, followed by metabolite annotation using annotation tools (e.g., database look ups or annotation tools) and then applying pathway analysis tools (e.g., ORA or TA) to determine the pathway activities. A pathway is assumed active when biological and environmental factors lead to the production of some or all of its metabolic products. In some cases, metabolite annotation is skipped, and statistical pathway activity is computed directly from measurements. In contrast, our inference-based approach utilizes a generative model (Figure 2B) that mimics biological processes inherent to the sample under study. Our presumed biological process assumes that when pathways are active, they cause the presence of some its metabolites, which in turn results in observations of masses through untargeted metabolomics.

PUMA first constructs a graphical model [28] that captures the complex relation among pathway activities, metabolites, and measurements in a single integrated model. The model produces values that are observed (measured), as well as hidden variables of interest, which cannot be directly observed but rather inferred from those values that can be observed. In our case, the observations correspond to mass measurements collected through untargeted metabolomics. The hidden variables are pathway activities and the presence of a metabolite in a biological sample.

Our generative model assumes the following biological process: one or more pathways are active. An active pathway causes the presence of some of its metabolites, which in turn results in observations of masses through untargeted metabolomics data collection. The generative model also assumes that the mass spectrometer is not biased towards particular measurements or classes of molecules. The lack of observations regarding the presence of masses therefore contributes some evidence regarding the corresponding pathway activity. The generative model is parameterized with prior information, or prior probabilities, about the behavior of the biological process. Here, we provide priors on each step in the biological process: for pathway activities, on pathways generating their metabolites, and metabolites mapping to mass measurements. We assume that the biological sample has a metabolic model with I pathways, J metabolites, and K unique metabolite masses. A metabolite may have membership in one or more pathways. PUMA assumes prior knowledge of adducts and in-source fragmentation and utilizes the adjusted measured masses when mapping the measurements to model metabolites. An (adjusted) measured mass may be associated with one or more masses of the model metabolites. Masses of the model metabolites are mapped to discretized bins, where each bin is centered at a unique mass value and allows for a mass tolerance of ±15 ppm. Each model metabolite is assigned to a single bin that is centered closest to the metabolite’s mass. A binary vector w has K entries and indicates putative mass observations of metabolites in the model. An entry of 1 for $w_{k}$ in vector w indicates the observation (measurement) of at least one metabolite in the *k*^th^ bin while a 0 indicates no observation for any metabolite in that bin.

Let $a=\left(a_{i}:i=1,\ldots ,I\right)$ denote the status of I pathways in the biological sample, so a is a vector of binary random variables, where a value of 1 indicates that the corresponding pathway is active and 0 indicates inactivity. We assume that the $a_{i}$ random variables are independent, with a Bernoulli(λ) prior:

(1) $$p\left(a_{i}=1\right)=\lambda ,\;\;i=1,\;\ldots ,\;I$$

For simplicity in defining our model, we assume that λ is a model parameter and set it to a constant. As an alternative, we can give it a Beta prior.

Matrix Z is defined with I rows and J columns. Each entry $z_{ij}$ corresponds to the activeness of metabolite j in pathway i, where a value of 1 indicates metabolite j is active due to pathway i and a value of 0 indicates that metabolite j is not produced by pathway i. If a metabolite j is on a pathway i, then the metabolite is produced according to the following probability:

(2) $$p\left(z_{ij}=1|a_{i}=1\right)=\mu ,\;p\left(z_{ij}=1|a_{i}=0\right)=0$$

Otherwise, $p\left(z_{ij}=1|a_{i}\right)=0$ when j is not on i. For simplicity, we assume that all metabolites are equally likely to be generated with probability μ within an active pathway. Vector m collapses the matrix Z into a binary vector with J elements, indicating the activeness of a metabolite due to whichever pathway:

(3) $$m_{j}=\left[\underset{i}{\sum }z_{ij}>0\right]$$

Here [⋅] gives 1 when the condition inside is true or 0 otherwise.

As not all masses can be captured using the mass spectrometer, its observed accuracy is defined using parameter γ. Let $J_{k}$ define the group of metabolites that have masses in the k-th bin, then:

(4) $$p\left(w_{k}=0|m_{J_{k}}\right)={\left(1-\gamma \right)}^{\sum_{j\in J_{k}}m_{j}}$$

**Figure 3:**
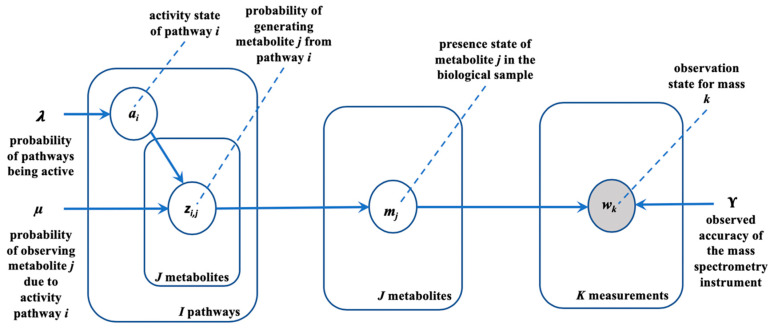
Graphical representation of the generative model. To avoid representing all I pathways, J metabolites, and K masses in the graph we use the ‘plate’ notation and draw one representative node per variable and enclosing these variables in a plate (rectangular box). The number of instances of each enclosed variable is indicated by the fixed constant in the lower right corner of the box. Random variables of the model (**a, z, m, w**) are shown in white circles. The variable **m** has a deterministic relationship with **Z**. The shaded circle, labelled **w**, represents an observed random variable. μ, λ, γ are parameters to the model.

This probability means that every metabolite present in the biological sample has a chance γ to be detected. In the case when all metabolites in $J_{k}$ are not observed ($\underset{j\in J_{k}}{\sum }m_{j}=0$), then mass k will not be observed. The detection of a metabolite is independent of the detection of others in the sample. No two groups, $J_{k}$ and $J_{k’}$, intersects because a metabolite has only one mass. The described model is described using the plate representation [29] (Figure 3). The model presents the joint probability distribution of random variables a, z, m and w defined as:

(5) p(a,z,m,w)=p(a;λ) p(z|a;μ) p(m|z) p(w|m;γ)

### 2.3. Inference

Using the probabilistic model, we infer pathway activities and metabolite presence from mass measurements. Specifically, we calculate the following probabilities. For each pathway i in the biological sample we calculate $p\left(a_{i}|w\right)$, the posterior probability of pathway i being active given evidence in mass measurements. PUMA utilizes Gibbs sampling to perform Bayesian inference [26] to approximate the posterior probabilities of pathway activities conditioned on the measurements. We then infer the presence of metabolites by calculating the posterior $p\left(m_{j}|w\right)$ for all j. We use the latter probabilities to rank a candidate set of metabolites for each mass measurement, where a candidate set provides one or more suggestion of chemical identities that have the same mass, within an error margin, as the observed one.

#### 2.3.1. Inferring Pathway Activities

Gibbs sampling is employed to perform Bayesian inference to approximate p(a|w), the posterior probability of pathway activities conditioned on the measurements. Naively sampling random variables *a* and *Z*, is time consuming. To speed the Gibbs sampler, we marginalize hidden variables Z. From the Bayesian formula:

(6) p(a|w)=p(w|a)p(a)/p(w)

Gibbs sampling is convenient in that there is no need to compute the denominator p(w) to draw samples from the posterior p(a|w). We only need to focus the computation of p(w|a) and p(a), where the latter was already assumed to have a Bernoulli distribution. Below we show how to compute p(w|a). We point out that p(w|a) decomposes as follows:

(7) $$p\left(w|a\right)=\prod_{k}p\left(w_{k}|a\right)$$

This is because metabolites in separate $J_{k}$ groups are independent given a, so do masses that are computed within these groups. Then we focus on the calculation of $p\left(w_{k}|a\right)$. Let $\phi_{j}\left(a\right)$ be the probability that at least one pathway in the biological sample generates metabolite $m_{j}$. That is, $\phi_{j}\left(a\right)=p\left(m_{j}=1|a\right)$. The detailed calculation of $\phi_{j}\left(a\right)$ is provided in Supplementary File S1, the calculation of $\phi_{j}\left(a\right)$ is:

(8) $$\phi_{j}\left(a\right)=1-{\left(1-\mu \right)}^{n_{j}}$$

with $n_{j}$ being the number of active pathways that $m_{j}$ is on. Probability $p\left(w_{k}|a\right)$ is then computed as follows:

(9) $$p\left(w_{k}|a\right)=\{\begin{array}{ll} 1-\Pi_{j\in J_{k}}\left[1-\gamma \phi_{j}\right] & w_{k}=1\\ \Pi_{j\in J_{k}}\left[1-\gamma \phi_{j}\right] & w_{k}=0 \end{array}$$

The expression $1-\gamma \phi_{j}$, a number between 0 and 1, represents the likelihood that the mass spectrometer did not measure the activity of metabolite $m_{j}$. Combining $p\left(w_{k}|a\right)$ with the Bernoulli prior p(a), we have the joint probability *p*(w,a), which is sufficient for running the sampler and getting samples from the posterior. If λ has a Beta prior, then we will sample *a* and λ together from p(λ)p(a|λ)p(w|a).

#### 2.3.2. Inferring Metabolite Annotations

With samples drawn from p(a|w), we approximate $p\left(m_{j}|w\right)$, the posterior probability distribution of metabolite j being present in the biological sample. Instead of running the Gibbs sampling procedure again, we use previously collected samples of a from p(a|w) to estimate the probability $p\left(m_{j}|w\right)$. Let S={a ∈samples of p(a|w)} be a set of samples from the distribution p(a|w), then:

(10) $$p\left(m_{j}|w\right)=\Sigma_{a}p\left(m_{j},a|w\right)=\Sigma_{a}p\left(m_{j}|a,w\right)p\left(a|w\right)\approx \frac{1}{\left|S\right|}\;\Sigma_{a\in S}\;p\left(m_{j}|a,\;w\right)$$

The probability $p\left(m_{j}|a,\;w\right)$ has efficient computation. Let $k_{j}$ denote the entry of w corresponding to metabolite j, and let $\backslash k_{j}$ denote other entries in w. Then:

(11) $$p\left(m_{j}|a,\;w\right)=\frac{p\left(m_{j},\;w|a\right)}{p\left(w|a\right)}=\frac{p\left(m_{j},\;w_{k_{j}}|a\right)p\left(w_{\backslash k_{j}}|a\right)}{p\left(w|a\right)}$$

Here we use the fact that $m_{j}$ and $w_{k_{j}}$ are independent of other mass observations when a is given. With this relation, we have:

(12) $$p\left(m_{j}=1|a,\;w\right)=\frac{p\left(m_{j}=1,\;w_{k_{j}}|a\right)}{p\left(m_{j}=1,\;w_{k_{j}}|a\right)+p\left(m_{j}=0,\;w_{k_{j}}|a\right)}$$

Here, the terms that are constants to $m_{j}$ are canceled. Finally, we can compute $p\left(m_{j},w_{k_{j}}|a\right)$ by marginalizing over all $m_{j},$ for $j^{\prime }\neq j$ and $j^{\prime }\in J_{k}$:

(13) $$\begin{array}{l} p\left(m_{j},\;w_{k_{j}}|a\right)=\Sigma_{m_{J_{k}\backslash j}}\;p(m_{j},\;m_{J_{k}\backslash j},\;w_{k_{j}}|a)\\ \;\;\;\;\;\;=\Sigma_{m_{J_{k}\backslash j}}\;p\left(w_{k_{j}}|m_{j}\;,\;m_{J_{k}\backslash j}\right)p\left(m_{j},\;m_{J_{k}\backslash j}|a\right) \end{array}$$

We decompose the above formulation into two terms for managing calculations. These two terms, $p\left(w_{k}|m_{J}{}_{k}\right)$ and $p\left(m_{j},\;m_{J_{k}\backslash j}|a\right)$ are further derived and re-expressed in the Supplementary Material, to yield the following:

(14) $$p\left(m_{j},\;w_{k_{j}}|a\right)=\{\begin{array}{cc} \left(1-\phi_{j}\right)\left(\underset{j^{\prime }\in J_{k},\;j^{\prime }\neq j}{\prod }\left(1-\gamma \phi_{j^{\prime }}\right)\right) & m_{j}=0,\;w_{k_{j}}=0\\ \left(1-\phi_{j}\right)\left(1-\underset{j^{\prime }\in J_{k},\;j^{\prime }\neq j}{\prod }\left(1-\gamma \phi_{j^{\prime }}\right)\right) & m_{j}=0,\;w_{k_{j}}=1\\ \phi_{j}\left(1-\gamma \right)\underset{j^{\prime }\in J_{k},\;j^{\prime }\neq j}{\prod }\left(1-\gamma \phi_{j^{\prime }}\right) & m_{j}=1,\;w_{k_{j}}=0\\ \phi_{j}\left(1-\left(1-\gamma \right)\underset{j^{\prime }\in J_{k},\;j^{\prime }\neq j}{\prod }\left(1-\gamma \phi_{j^{\prime }}\right)\right) & m_{j}=1,\;w_{k_{j}}=1 \end{array}$$

We use these equations to calculate the probabilities $p\left(m_{j}=1,\;w_{k_{j}}|a\right)$ and $p\left(m_{j}=0,\;w_{k_{j}}|a\right)$. By normalizing the two terms to have a sum of 1, we get the posterior of metabolite annotations. The derived probabilities are used as a scoring metric to rank a candidate set for each mass measurement. Details on the derivation and implementation of metabolite annotation are provided in Supplementary File S1.

### 2.4. Implementation and Parameter Initialization

We implemented PUMA using PyMC3 [30], a probabilistic programming framework that allows for automatic Bayesian inference on user-defined models. In the implementation, we assume that λ has a β prior with parameters α=β=1. We sample both random variables a and λ. To draw samples from a posterior distribution, PyMC3 utilizes a Markov Chain Monte Carlo (MCMC) sampling technique [31]. The generative model was derived from the metabolic model for each of our case studies. The observed accuracy of the mass spec, γ, is assumed to be 0.9. Each entry in *μ* is assumed to be 0.5 if metabolite *j* exists on pathway i. *T,* the number of samples to draw from the model, is a variable that can be set in PyMC3. The sampler was run multiple times with T values equal to 500, 1000, and 1500. We assumed 100 burn-in samples. For all reported runs, increasing the number of drawn samples altered the computed probabilities slightly but did not affect the list of active pathways, based on a 0.5 activity threshold. Drawing 1000 samples was used as a default.

## 3. Results

### 3.1. Model Validation

To give confidence in the performance of PUMA, it is desirable to validate the generative models against a “ground truth” dataset, where all measured metabolites are annotated and there is sufficient experimental evidence to allow attributing measured metabolites to specific pathways. Predictions by PUMA can then be compared against this ground truth. Despite several databases that catalogue various metabolomics datasets (e.g., MetaboLights [32], Metabolomics Workbench [33]), there are currently no untargeted metabolomics sets that are 100% annotated. Further, there are no datasets that allow attributing metabolites to specific pathways through experimental work. We therefore design datasets that serve as “ground truth” datasets when validating pathway analysis tools. The datasets are generated to mimic biological processes where genes within pathways work in concert and result in enzymatic activities that produce metabolites [34]. These metabolites are then observed via mass spectrometry. As central metabolism and network topology is conserved across many organisms [35], we generated the synthetic datasets for a representative organism, the CHO cell, a popular biological sample that is discussed herein as a case study. Its cellular pathways and their metabolites are used as the basis for generating pathway and metabolite activities.

To model a variety of plausible cellular activity, several synthetic datasets are generated. The portion of active pathways and the portion of active metabolites are varied for each dataset. A random portion (0.3, 0.5, and 0.7) of pathways are assumed active, and a random portion (0.05, 0.10, 0.15, 0.20, 0.25, 0.50, 0.75) of metabolites within each active pathway are generated. For each portion of active pathways and for each portion of active metabolites, 100 metabolomics datasets reflecting the masses of the active metabolites were generated. The observed accuracy γ was set to 1.

**Figure 4:**
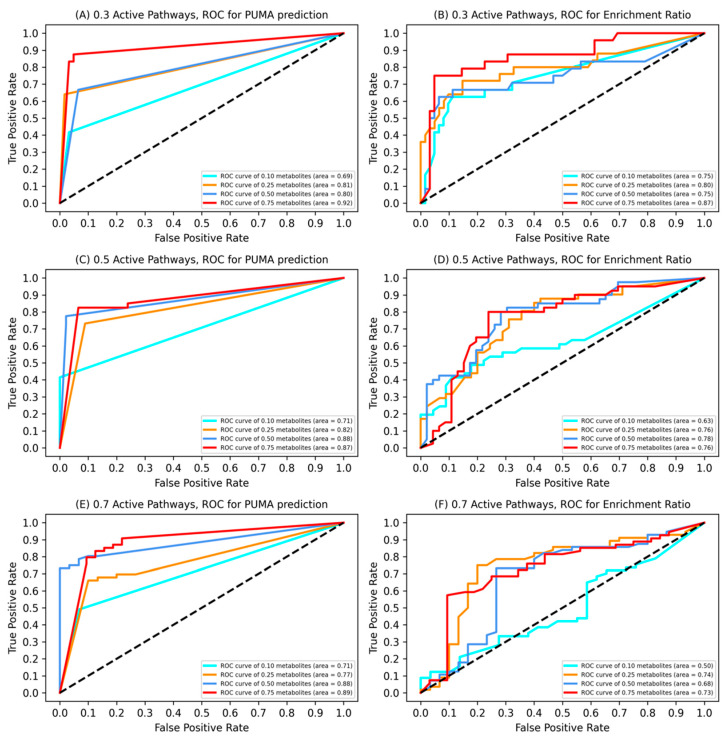
AUC for ROC curves for the synthetic datasets under different assumptions regarding pathway activity and metabolite generation. (**A**,**C**,**E**) PUMA. (**B**,**D,F**) Enrichment ratio.

We compared the likelihood of pathway activities computed by PUMA against the enrichment ratios for the synthetic datasets. The enrichment ratio for a particular pathway is defined as the ratio of measured masses that map to metabolites within the pathway to its size. We use AUC, the area under the Receiver Operating Characteristic (ROC) curve to report the results. A ROC curve plots TPR (True Positive Rate) vs. FPR (False Positive Rate) at different classification thresholds. The AUC considers the performance of a classifier across all possible classification thresholds. Lowering the threshold for classifying a pathway as active results in classifying more pathways as active, thus increasing both false positives and true positives. The AUC effectively reports on the probability that the method ranks an active pathway that is selected randomly more highly than an inactive pathway that is selected randomly. The ROC curves are plotted for PUMA and for enrichment ratios (Figure 4). The AUCs are consistently higher for PUMA than for the enrichment ratios, with the exception of the case when pathway activities are low (0.3) and only 0.1 of metabolites within each pathway are produced. An increase in the number of measurements while the pathway activity is fixed provides PUMA with more evidence and in general results in PUMA performance improvements that are more pronounced than for enrichment analysis. The lowest AUC for PUMA occurs with the lowest pathway activity and lowest number of generated metabolites (AUC = 0.69), while the lowest AUC for the enrichment ratio analysis occurs with the highest portion of active pathways, and smallest number of generated metabolites (AUC = 0.50). Importantly, PUMA outperformed the enrichment ratio, on average, by 8%, with average AUCs of 0.81 and 0.73 for PUMA and enrichment ratios, respectively.

We then applied PUMA to each dataset and averaged PUMA’s precision, recall and accuracy on identifying the presumed active pathways. At a pathway activity of 0.3 (Figure S1A), as we have more observed metabolites, recall increases because PUMA has more evidence in terms of observations to recover the correct pathway activities. Precision, PUMA’s ability to label true positives correctly, is greater than 0.71, regardless of the active fraction of metabolites. Accuracy improves with increased active metabolites due to the corresponding increase in PUMA’s ability to identify true positives. This trend holds for other assumptions about pathway activities (Figures S2A and S3A).

We investigate how uncertainty in metabolite annotation impacts inference regarding pathway activity. Before running PUMA, each mass measurement is attributed to a presumed active metabolite, thus removing annotation uncertainty. Results (Figures S1B, S2B, and S3B) show a similar trend to those in Figures S1A, S2A, and S3A. A similar trend holds when each measured mas is randomly assigned a metabolite amongst model metabolites with the same mass as a measured mass (Figures S1C, S2C, and S3C). This result emphasizes that computing pathway activities without the explicit step of performing metabolite annotation via spectral databases or annotation tools is a profitable approach. PUMA can, therefore, be used to accelerate the process of pathway activity analysis by direct use of mass measurements and bypassing metabolite annotation using spectral databases.

We further investigated the robustness of the model to its parameters. While prior runs assumed that the probability of observing a metabolite due to a particular pathway activity was 0.5, we varied the corresponding model parameter μ to 0.25 and to 0.75 and re-ran PUMA. The results (Figure S4) show that inference is dominated by other aspects of the model and that inference is robust to this model parameter.

### 3.2. Case Study: Chinese Hamster Ovary (CHO) Cell

We apply PUMA to LC-MS (Liquid-Chromatography Mass Spectrometry) metabolomics data for CHO cell cultures belonging to a low growth cell line [15] (Supplementary Table S1). LC-MS data was collected under three different combinations of liquid chromatography methods and positive or negative ionization modes. The authors processed the data using the CAMERA tool and the dataset was limited to masses corresponding to ions formed through protonation or deprotonation. When combined, the data provides a more comprehensive characterization of the sample in the form of 8711 measurements. The metabolic model for the CHO cell was extracted from KEGG [36], based on metabolites and pathways for the cricetulus griseus (Chinese hamster) under organism code *cge*. The model had 86 pathways, 1534 metabolites, and 722 unique mass measurements. The *m*/*z* of the precursor ions in the measurement datasets were adjusted based on the ionization mode by adding or subtracting the mass of one proton. Adjusted measurements ± a 15 ppm are then used to initialize the observation vector w for each dataset, as described in Section 2.2. This model and LC-MS dataset were used prior to evaluate BioCAn [15], a tool that aggregates results from spectra databases, annotation tools and network connectivity. The BioCAn evaluation thus provided putative identities in the form of KEGG identities, and also provided annotations using METLIN and HMDB. We, therefore, compare metabolite annotations with those provided for METLIN, HMDB, and BioCAn.

#### 3.2.1. Probabilities of Pathway Activities

Detailed results for each dataset and for the combined data set is provided in Supplementary Table S1. A pathway is considered active if $p\left(a_{i}|w\right)$ is equal to or greater than 0.5. As mass observations differ from one set of measurements to another, the predicted activity differs among the datasets. A detailed discussion of the results for the individual datasets is provided in Supplementary File S1. The rest of the CHO cell analysis provided here is based on the combined dataset.

Many of the 42 pathways identified active by PUMA are biologically relevant. The biological activity of most pathways such as TCA cycle, essential for energy metabolism, Biotin (vitamin B7) metabolism, amino acid synthesis, and many others, is expected. However, the activity of some pathways, including caffeine and drug pathways, is biologically unlikely active in the CHO cell samples. Based on our experiments using the synthetic datasets, we expect some PUMA predictions to be false.

**Figure 5:**
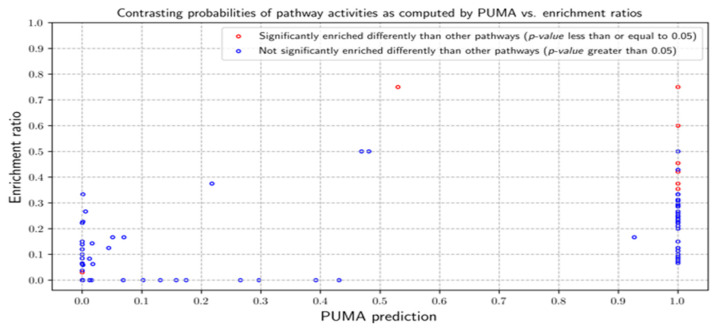
Probability of pathway activities as computed by PUMA vs. enrichment ratios for CHO cell. Each data point is marked as either statistically enriched (red) or non-statistically enriched (blue) based on a Fisher’s Exact Test *p*-values of 0.05.

Pathway activities predicted by PUMA are contrasted against pathway enrichment ratios (Figure 5). Pathways are labeled as *statistically enriched* based on statistical significance of their ratios using Fisher’s Exact Test (FET). The null hypothesis is that there is no difference between the enrichment ratios of pathways in the sample. A *p*-value equal to or less than 0.05 is considered significant. Eight pathways are designated statistically enriched. These pathways are galactose metabolism, fatty acid degradation, purine metabolism, N-glycan biosynthesis, amino sugar and nucleotide sugar metabolism, glycosaminoglycan degradation, glycerophospholipid metabolism, lipoic acid metabolism. Among them, six pathways were predicted by PUMA to be active with probability equal to 1 while the N-Glycan biosynthesis pathway had a 0.53 likelihood of being active. Fatty acid degradation is predicted to be inactive. There were many pathways that had low enrichment ratios and low PUMA-predicted activity.

While there was consensus in some cases, there were also differences. PUMA designates some pathways as active despite low enrichment ratios. For example, the enrichment ratios of the TCA cycle, fatty acid biosynthesis, ubiquinone and terpenoid-quinone biosynthesis are 0.15, 0.29, and 0.13, respectively. Meanwhile, PUMA predicted these pathways active with a likelihood of 1. There are three pathways with enrichment ratio equal to 0.5. Of them, one pathway, biotin metabolism, is assigned active by PUMA with probability 1.0. The biotin metabolism pathway has a measured mass that is unique and cannot be generated by other pathways. However, the other two pathways, both glycosphingolipid biosynthesis pathways, are predicted active with probability less than 0.5 (0.47 and 0.48). The reason was as follows: the observed mass measurements in the glycosphingolipid biosynthesis pathways could be mapped to galactose metabolism and glycosaminoglycan degradation pathways that are associated with a unique measurement that cannot be attributed to any other pathway in model (similar to the case of w_5_ in our illustrative example Figure 1). As the result, the glycosphingolipid biosynthesis pathways were assigned probabilities less than 0.5, while the pathways with the unique measurements are predicted active with high probability.

#### 3.2.2. Probabilities of Metabolite Annotations

A particular measurement was associated with a model metabolite if its mass matched the measured mass within the bin tolerance. Each measurement therefore may be assigned zero, one or more possible annotation. Probabilities of each metabolite being present in the sample as inferred by PUMA are used to score and rank the putative annotations. Here, the top ranked metabolite(s) for each mass is considered as the *PUMA candidate set.*

**Figure 6:**
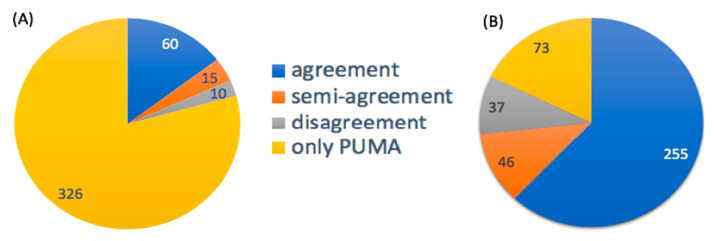
Metabolite annotations attained with PUMA against those identified by: (**A**) searching spectral databases, HMDB and METLIN, and (**B**) BioCAn. The blue slice in each pie represents “agreement”. The orange and gray slices represent “semi-agreement” and “disagreement” respectively. Finally, the yellow slice represents the number of mass measurements that could only be annotated by PUMA.

We assess the accuracy of PUMA annotations by comparing the level of agreement of PUMA annotations with those using two other techniques, spectral database searches and BioCAn (Figure 6). Spectral signatures collected for the CHO Cell were looked up in METLIN and HMDB as was previously reported [15]. Out of 411 mass measurements, 85 were matched using their spectral signatures either in HMDB or METLIN. Each such measurement had one or more chemical identities assigned to the spectral signature. Here, the highest scoring metabolite(s) for each measurement using METLIN and HMDB formed the *spectral database candidate set*.

For each measurement, the PUMA candidate set was compared against the spectral database candidate set. The comparison leads to four different scenarios: “agreement”, “semi-agreement”, “disagreement”, and “only PUMA”. An “agreement” scenario is where the PUMA candidate set exactly matches the spectral database candidate set. Such agreement occurs in 60 cases. A “semi-agreement” is when the spectral database candidate set is a subset of the PUMA candidate set. That is, unlike the “agreement” scenario, there is not complete consensus regarding the top candidate set, and hence the “semi-agreement” label. There are 15 cases of “semi-agreement”. A “disagreement” scenario is when the spectral database candidate set does not overlap with the PUMA candidate set. There were 10 such cases of “disagreement”. In 7 such cases, the spectral database candidate set is the second likely putative annotation identified by PUMA. These putative annotations, which were not part of the PUMA candidate set, had high PUMA activity scores that were close in value to scores of the metabolite(s) in the PUMA candidate set. In the remaining 3 cases, however, the spectral database candidate is assigned a low score by PUMA. Clearly PUMA and METLIN/HMDB disagree in regard to these three cases. An “only PUMA” scenario is when the spectral database candidate set was empty, but the PUMA candidate set was not. There were 326 such cases. This large number of “only PUMA” cases reflects the low coverage of spectral databases.

PUMA annotations are compared against those obtained using BioCAn [15]. BioCAn aggregates results from spectral database searches and in silico fragmentation tools. Further, BioCAn estimates the confidence in annotation not only based on consensus scoring but also based on the presence of metabolites that are connected to the mass measurement through substrate-product relationships. BioCAn identifies 338 out of 411 mass measurements that are annotated by PUMA. The top ranked metabolite(s) for each mass as annotated by BioCAn is considered the “BioCAn candidate set”. We analyze various scenarios as we did when comparing against the spectral database candidate set. Again, there were four scenarios: “agreement”, “semi-agreement”, “disagreement”, and “only PUMA”. The definitions of these scenarios are similar to the ones provided for the METLIN/HMDB comparison, but against the BioCAn candidate set instead of the spectral database candidate set. There are 255 cases of “agreement”, 46 cases of “semi-agreement”, 37 cases of “disagreement”, and 73 “only PUMA” annotations. The disagreements fell into two categories. In 17 out of 37 cases, there was disagreement regarding the top candidate, where PUMA ranked BioCAn’s candidate as second best. There were genuine disagreements in 20 cases where the annotation by BioCAn was assigned a low score by PUMA.

To validate annotations, BioCAn experimentally validated 50 of their predicted annotations against chemical standards. A subset of 37 compounds were confirmed present based on the standards. Thus, BioCAn’s precision on this 50-compound dataset is 0.740 (37/50). PUMA correctly calculated the likelihood for 33 of the 50 compounds in the sample: 25 were true positives, and 8 were true negatives. PUMA miscalculated the likelihood for 17 annotations: 5 were false positives and 12 were false negatives. PUMA’s precision for this 50-compound dataset is 0.833, thus achieving a significant additional 0.093 improvement in precision over BioCAn. Recall for PUMA for this 50-compound dataset was 0.676.

In summary, comparing PUMA annotations against those obtained through spectral database and BioCAn shows significant levels of agreement. METLIN, HMDB and BioCAn incorporate spectra signatures during annotation while PUMA relies solely on pathway organization and mass measurements. Importantly, for the CHO cell, PUMA increased annotation by 383% over spectral databases and by 21% over BioCAn.

#### 3.2.3. Evaluation of PUMA in Overcoming Uncertainty in Annotation

As a measured mass may be attributed to more than one metabolite, there is uncertainty inherent in mapping measurements to metabolites. Matrix τ, with *J* rows and *K* columns, maps metabolites in the model to their corresponding masses. We investigate if PUMA benefits from reducing the mapping uncertainty by adjusting the τ matrix prior to running PUMA. In the case of the CHO cell, the annotations obtained via spectral lookups in the METLIN and HMDB databases are used to assign identities to some of the measured masses prior to running PUMA. To capture this knowledge, matrix τ is modified to map each mass *k* to precisely a single metabolite *j*, which is identified based on the METLIN and HMDB annotations. Column entries other than $\tau_{\;i,\;j}$ are set to zero, indicating that mass *k* uniquely maps to metabolite j. Using the updated τ, PUMA calculated posteriors for pathway activities. There was a slight change in predicted posteriors (average increase of 0.003) compared to those obtained using the original τ matrix. The change however does not alter posterior probabilities sufficiently to modify the list of active pathways. We repeated the analysis but incorporated the annotation data available from BioCAn instead of that obtained through spectral databases. The change in τ caused a slight change in predicted posteriors (an average of 0.001 per pathway) compared to those obtained using the original τ matrix. The one significant change was for pathway Phenylalanine metabolism where pathway activity changed from 0.03 to 1.0. The phenylalanine metabolism pathway is responsible for producing tyrosine. This finding suggests that substantial additional annotations, as provided in the form of added annotations by BioCAn over the use of spectral databases, are required to inform inference in regard to pathway activities. 

### 3.3. Case Study: Human Urinary Sample

We apply PUMA to untargeted metabolomics datasets collected for human urinary samples analyzed by Roux et al. [27]. Detailed annotations using KEGG identities, if available, are provided for 384 metabolites based on careful analysis of 659 and 825 annotated ions in the positive and negative modes, respectively. As PUMA utilizes post-processed metabolomics data, we utilized the 384 mass measurements, which were already adjusted for ionization, adducts, and in-source fragment ions, as input to PUMA. Byproducts in the urine may be attributed to human metabolic pathways. We, therefore, modeled pathways responsible for these byproducts using the human metabolic model from MetaCyc [37]. The model had 275 pathways, 716 metabolites, and 565 unique masses. To evaluate PUMA without prior knowledge of metabolite annotations, each of the 384 mass measurements were mapped to all model metabolites that were within ±15 ppm, as described in Section 2.2. Only 123 out of the Roux et al. measured masses matched to metabolites in the model.

#### 3.3.1. Probabilities of Pathway Activities

**Figure 7:**
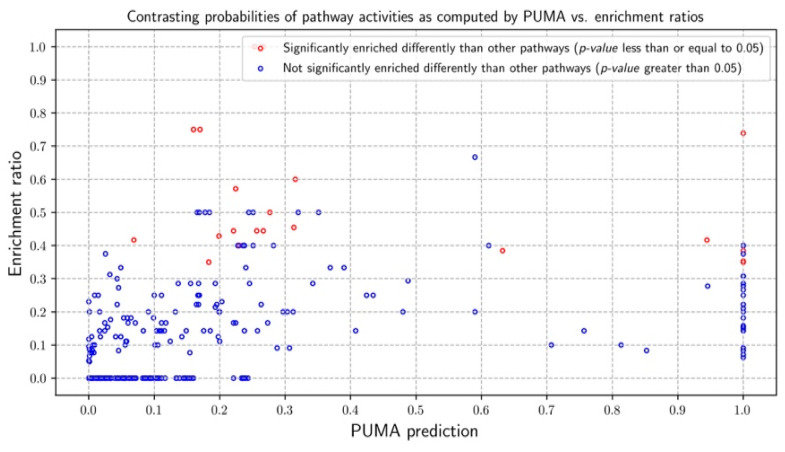
Probability of pathway activities as computed by PUMA vs. enrichment ratios for the human urine sample. Each data point is marked as either statistically enriched (red) or non-statistically enriched (blue) based on a Fisher’s Exact Test *p*-values of 0.05.

PUMA designated 41 pathways as active in human urinary sample (Supplementary Table S1). We investigate how inference results compare with pathway enrichment ratios (Figure 7). Of the 41 pathways designated to be active using PUMA, six pathways (tRNA charging, 4-hydroxyproline degradation I, histidine degradation VI, lysine degradation II, purine ribonucleosides degradation to ribose-1-phosphate, nicotine degradation III) are statistically enriched. As in the CHO cell cases, there were cases of agreement and disagreement. There are several pathways were PUMA predicts low activity, while enrichment assumes a high enrichment ratio, including alanine biosynthesis II, glutamate degradation II, aspartate biosynthesis, arginine degradation VI, and alanine degradation III. The probabilities for these pathways are 0.26, 0.22, 0.17, 0.31, and 0.25, respectively, while the corresponding enrichment ratios are 1.0, 0.57, 0.75, 0.6, and 1.0. Many measurements assigned to these pathways, however, are not unique as they can generated due to activity of other pathways.

#### 3.3.2. Probabilities of Metabolite Annotations

**Figure 8:**
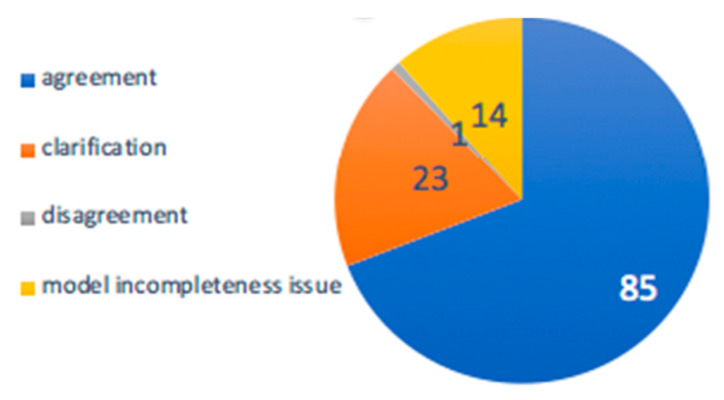
Metabolite annotations attained with PUMA against those identified by Roux et al. The blue slice represents “agreement”. The orange slice represents “clarification”. The gray slice represents “disagreement” and the yellow slice represents “model incompleteness issue”.

The PUMA probabilities for each metabolite being present in the sample are used to score and rank metabolites. Only the top ranked metabolite(s) for each mass are considered as the *PUMA candidate set*. We compared our annotation against those provided by Roux et al. [27] (Figure 8). These annotations were either identified by matching at least two of their physicochemical parameters to those in a reference standard or annotated through spectral database lookups (HMDB). Some measurements were annotated as isomers, without identifying the precise chemical molecular identity. Each measurement was assigned the best possible match. We refer to this match as the *Roux candidate*. When comparing the PUMA candidate set to the Roux candidate, there were four scenarios: “agreement”, “clarification”, “disagreement”, and “model incompleteness issues”. An “agreement” scenario is where the PUMA candidate set exactly matches the Roux candidate. Such agreement occurs in 85 cases. A “clarification” scenario is when the PUMA candidate set provided a specific chemical annotation while the Roux candidate annotated the measurement as an isomer. There were 23 cases of “clarification”. A “disagreement” scenario is when the Roux candidate was available in the model but was not predicted as match by PUMA. There was one case of “disagreement”. A “model incompleteness issues” scenario is when the Roux candidate was not in the model, reflecting that PUMA provides the best match within the scope of model metabolites. There were 14 “model incompleteness issue”. More comprehensive metabolic model could address such issues.

### 3.4. Model Convergence, Complexity, and Runtimes

The time and space complexity in sampling the model is *O*(T × I×J). The runtime for drawing 1000 samples for pathway activity prediction and metabolite annotation for the CHO cell dataset were 231 and 0.5 s, respectively. The corresponding runtimes for the Human Urinary case study were 280 and 0.4 s, respectively. The runs were performed on a Dell PowerEdge R815 server with 64 cores (4× AMD Opteron 6380 processors) and 128 GB of RAM, running at 2.5 GHz.

## 4. Discussion and Conclusions

We presented in this paper PUMA, a probabilistic approach to interpret mass measurements collected through untargeted metabolomics. PUMA first uses inference to determine pathway activities. While prior works focused on computing pathway enrichment in the context of comparing one sample against the other, here, we define a pathway as active based on its likelihood of being responsible for the presence of one or more metabolomics measurements. In determining activity, PUMA reasons about the complex relationships between the measurements as well as known pathway as defined through the underlying biochemical networks. In doing so, levels of uncertainty in mapping measurements to metabolites and pathways are significantly reduced. Moreover, a clearer view of the likelihood of pathway activity levels emerges when compared to simple enrichment analysis. PUMA then utilizes the likelihood of pathway activities to compute the posterior probability distribution of metabolites being present in the sample. The mathematical formulation for computing pathway activities and metabolite annotation probabilities was essential in achieving fast runtimes. For each test case, PUMA’s runtime for calculating pathway activities was under five minutes. The runtime for calculating metabolite annotation was less than a second. A naïve implementation of pathway activity inference would have caused significantly longer runtimes.

PUMA was validated using synthetic datasets, generated to compensate for the lack of “ground truth” metabolomics datasets where all metabolites and pathway activities are known. As expected, additional measurements consistently improve PUMA recall scores. This study provided two strong results. First, fixing identities of the measurements prior to computing pathway activities provide limited improvement in PUMA performance, thus emphasizing that bypassing metabolite identification prior to computing pathway activity is a valid approach for determining pathway activities. Second, using AUC for the ROC curve as a metric, the pathway activity likelihoods computed using PUMA, on average, outperform the pathway enrichment analysis by an average of 8%.

PUMA was applied to two case studies, the CHO test case and the human urine test case. In regard to pathway analysis, PUMA identifies pathways that have a high likelihood of being active but have statistically low enrichment ratios, and pathways with low likelihood of being active yet with statistically high enrichment ratios. PUMA, therefore, offers a perspective on pathway activity that is distinctly different from that offered by statistical enrichment.

In regard to metabolite annotations, PUMA results had high agreement to prior annotations. This high level of agreement occurs despite the fact that PUMA does not utilize additional information in form of spectra signatures, as employed by other techniques. In the case of the CHO cell test case, PUMA increased the percentage of mass annotation by 383% over spectral lookups and by 21% over BioCAn. PUMA analysis of a 50-compound dataset that was experimentally verified by BioCAn, yielded 0.676 recall and 0.833 precision, which provides a significant 0.093 improvement in precision over BioCAn. For the human urine test case, PUMA showed agreement in annotating 85 metabolites that were annotated before using database looks ups. PUMA also suggested new putative identities for measurements that were previously identified only as isomers. Agreements demonstrated for both test cases against prior experimental annotations (those provided by BioCAn [15] and by Roux et al. [27]) provide strong evidence for PUMA’s annotation capabilities.

The approach of predicting differential functional activity directly from spectral features without a priori metabolite annotation was previously shown effective using Mummichog [22]. PUMA utilizes all measurements to compute the likelihood of pathway activities that gave rise to the measurements. PUMA considers a biological sample under a certain condition, while Mummichog utilizes differentially expressed measurements to determine differently observed pathways/modules. PUMA uses the likelihood of pathway activities to derive metabolite annotations. Analyzing both the synthetic data sets and the CHO cell test case, PUMA confirms that the organization of metabolic networks can resolve the ambiguity in metabolite annotation to a large extent, as previously noted when using Mummichog.

PUMA is based on inference but differs from other inference-based methods. ProbMetab [38] uses a probabilistic method [39] to assign empirical formulas to measured spectra given potential formulas. The method proposed by Jeong et al. constructs a generative model to infer the likelihood of a metabolite in the sample and the correctness of matching the measurement to a candidate metabolite within a spectral database based on measured spectra’s similarity to that of the proposed candidate and to other competing spectra in the database [40]. The competing spectra, however, may not be relevant to the sample. Del Carratore et al. uses evidence in the form of isotope patterns, adduct relationship and biochemical connections to infer metabolite annotations [41]. ZODIAC [42] also utilizes inference to re-rank molecular formula candidates suggested by SIRIUS [43].

The presented method herein provides a novel, efficient, inference-based pathway analysis technique. PUMA can be enhanced in multiple ways. PUMA assumes that pathway activities are independent. Under this assumption, observed (present or absent in the sample) metabolites are not independent because they share the same set of hidden random variables. This relationship is evident in the graphical representation of the generative model (Figure 3). However, PUMA does not consider biochemical dependencies between substrates and products. The generative model proposed herein can be strengthened by considering such relationships. Further, modeling instrument bias and sensitivity to metabolite concentrations can enhance PUMA, as well as other annotation tools. PUMA, like Mummichog, BioCAn, and other enrichment analysis tools depend on genome-scale metabolic models. However, such models are often incomplete, yielding incomplete analyses as evidenced in the scenario of “model incompleteness issues” when applying PUMA to the human urine sample. Integrating metabolomics data with genomics data can potentially yield improved metabolic models [44]. PUMA, like many other tools, can be enhanced by investigating instrument bias and sensitivity to metabolite concentrations. Herein, synthetic datasets served an important role in validating PUMA’s performance. The synthetic datasets were generated using the CHO cell as a model organism and assumed several scenarios with a wide range of pathway activity and observations. It is possible to make alternate assumptions regarding the data generation process and further adjust the generation assumptions to account for potential biases in collecting the measurements.

## Supplementary Materials

The following are available online at https://www.mdpi.com/2218-1989/10/5/183/s1. Supplementary File S1.pdf provides additional derivations in support of inference of pathway activity and metabolite annotations. Supplementary Table S1 provides additional results on the synthetic dataset and on the CHO case study.

## Availability

The code and datasets are on https://github.com/HassounLab/PUMA.
