# Supplementary material for: Pathway-Activity Likelihood Analysis and Metabolite Annotation for Untargeted Metabolomics Using Probabilistic Modeling
Source: Metabolites. 2020 May 3;10(5):183. doi: 10.3390/metabo10050183 (PMC7281100; doi:10.3390/metabo10050183)
Supplement: Supplementary file 1 [file metabolites-10-00183-s001.zip › Supplementary/PUMA_Supp_rev1.pdf]

Pathway Activity Analysis and Metabolite Annotation for Untargeted Metabolomics using Probabilistic Modeling

Ramtin Hosseini, Neda Hassanpour, Li-Ping Liu and Soha Hassoun
Department of Computer Science
Tufts University, Medford, MA

Supplementary File 1

1. Derivations in support of inferring pathway activities

We show the derivation of $\phi_j(a) = p(m_j = 1|a)$. We segment all z_{ij} random variables into groupings as they relate to metabolite j. The set of observation of metabolite j in pathways can be represented as a vector $[z_{1j}, z_{2j}, z_{3j}, \dots, z_{Ij}]$. The union of elements in this vector is denoted $o_{.j}$. If o_{ij} occurs, it implies that pathway i generates metabolite j. Note m_j = 0 is equivalent to the fact that z_{ij}-s for all i-s are zero. Furthermore, z_{ij}-s are independent when pathway activities a are known. Then:

$$p(m_j = 1|a) = 1 - \prod_{i=1,\dots,I} \mathrm{p}(z_{ij} = 0|a) = 1 - (1-\mu)^{n_j}$$

with $n_j = \sum_{j:j \text{ is on } i} a_i$ denotes the number of active pathways that contain metabolite j. Denote $\phi_j(a) = 1 - (1-\mu)^{n_j}$. Note that m_j-s are independent when a is given.

To calculate $p(w_k|a)$, we marginalize out metabolites m_{J_k}that have mass k.

$$p(w_k|a) = \Sigma_{m_{J_k}} p(w_k|m_{J_k}).\, p(m_{J_k}|a)$$

It is easier to do the marginalization for $w_k = 0$, which indicates no observation of mass k. The derivations for both cases are as follows:

$$p(w_k = 0|a) = \Sigma_{m_{J_k}} \Pi_{j\in J_k}[[(1-\gamma).\phi_j]^{m_j}(1-\phi_j)^{1-m_j}] = \Pi_{j\in J_k}[(1-\gamma)\phi_j + (1-\phi_j)]$$
$$= \Pi_{j\in J_k}[1-\gamma\phi_j]$$

$$p(w_k = 1|a) = 1 - p(w_k = 0|a) = 1 - \Pi_{j\in J_k}[1-\gamma\phi_j]$$

2. Derivations in support of inferring metabolite annotation

We have the following probability distribution for mass observation given set of metabolites:

$$p(w_k|m_{J_k}) = \begin{cases} 1 - \prod_{j\in J_k}(1-\gamma)^{m_j} & w_k = 1 \\ \prod_{j\in J_k}(1-\gamma)^{m_j} & w_k = 0 \end{cases}$$

We can divide m_{J_k} into two independent sets, $m_{J_{k+}}$ and $m_{J_{k-}}$, where the first set corresponds to all metabolites of mass k present in the sample, while the latter set corresponds to metabolites of mass k that are not present in the sample:

$$p(m_{J_k}|a) = p(m_{\mathrm{j\in J_{k+}}}, m_{\mathrm{\in J_{k-}}}|a) = \prod_{\mathrm{j\in J_{k+}}} \phi_j \ . \prod_{\mathrm{j\in J_{k-}}} (1-\phi_j) = \prod_{j\in \mathrm{J_k}} (1-\phi_j)^{1-m_j} (\phi_j)^{m_j}$$

We show the derivation of equation computing $p\left(m_j,\ w_{k_j}\middle|a\right)$ in the main manuscript for one case, $w_{k_j} = 1, m_j = 0$:

$$\Sigma_{\mathrm{m_{J_{k\backslash j}}}} p\left(w_{k_j} = 1\middle|m_j = 0, m_{J_k\backslash j}\right) p(m_j = 0, m_{J_k\backslash j}|a)$$

$$= \Sigma_{\mathrm{m_{J_{k\backslash j}}}} \left(1 - \prod_{j'\in J_k, j'\neq j} (1-\gamma)^{m_{j'}}\right)\left((1-\phi_j) \prod_{j'\in J_k, j'\neq j} (1-\phi_{j'})^{1-m_{j'}} (\phi_{j'})^{m_{j'}}\right)$$

$$= (1-\phi_j)\Sigma_{\mathrm{m_{J_{k\backslash j}}}} \left(\prod_{j'\in J_k, j'\neq j} (1-\phi_{j'})^{1-m_{j'}} (\phi_{j'})^{m_{j'}} - \prod_{j'\in J_k, j'\neq j} (1-\phi_{j'})^{1-m_{j'}} \left(\phi_{j'}(1-\gamma)\right)^{m_{j'}}\right)$$

$$= (1-\phi_j)\left(\sum_{m_{J_k\backslash j}} \prod_{j'\in J_k, j'\neq j} (1-\phi_{j'})^{1-m_{j'}} (\phi_{j'})^{m_{j'}} - \sum_{m_{J_k\backslash j}} \left(\prod_{j'\in J_k, j'\neq j} (1-\phi_{j'})^{1-m_{j'}} \left(\phi_{j'}(1-\gamma)\right)^{m_{j'}}\right)\right)$$

$$= (1-\phi_j)\left(\prod_{j'\in J_k, j'\neq j} \sum_{m_{j'}} (1-\phi_{j'})^{1-m_{j'}} (\phi_{j'})^{m_{j'}} - \prod_{j'\in J_k, j'\neq j} \sum_{m_{j'}} (1-\phi_{j'})^{1-m_{j'}} \left(\phi_{j'}(1-\gamma)\right)^{m_{j'}}\right)$$

$$= (1-\phi_j)\left(\prod_{j'\in J_k, j'\neq j} 1 - \prod_{j'\in J_k, j'\neq j} (1-\gamma\phi_{j'})\right)$$

$$= (1-\phi_j)\left(1 - \prod_{j'\in J_k, j'\neq j} (1-\gamma\phi_{j'})\right)$$

Other cases for w_{k_j} and m_j can be similarly derived.

3. Speeding up inference for metabolite annotation

To speed our implementation, we vectorize the matrix computations and deploy NumPy broadcasting. The output of pathway prediction activity is a matrix with S rows and I columns. Each row corresponds to the activity of pathways in the drawn sample. This matrix will be multiplied by a matrix with I rows and J columns. This matrix is defined to be sparse. By doing this, we will get a matrix with S rows and J columns which we call matrix C. Each element of this matrix corresponds to n_j. By creating these matrices, we can compute ϕ as:

$$\phi = 1 - \exp(\log(1-\mu) \,.\, C)$$

We define Φ to be a matrix with S row and J columns. We denote $\psi_j = \prod_{j' \in J_k,\, j' \neq j}(1 - \gamma\phi_{j'})$.

To calculate matrix ψ, first we create a matrix called B. Each element in B is the corresponding $\log(1-\gamma\phi_j)$. This matrix could be created by tweaking matrix C. We define matrix τ to implement the definition for J_k. This matrix has J rows and K columns and maps metabolites to their corresponding mass bin. By doing the following computations, we can compute ψ as:

$$\psi = B * \tau * \tau^T - B$$

$$V = W.\tau$$

The resulting closed form formula of the metabolite annotations are as follows:

$$R_0 = V * (1-\Phi) * (1-\exp(\psi)) + (1-V) * (1-\Phi) \qquad m_j = 0$$

$$R_1 = V * \Phi * (1-(1-\gamma) * \exp(\psi)) + (1-V) * \Phi * (1-\gamma) \qquad m_j = 1$$

Matrix η is defined with I row and J columns. Each entry η_{ij} indicates the presence/ absence of metabolite j on pathway i. By normalizing the posteriori likelihood of metabolite annotations, we get the following closed form expression:

$$p(m_j = 1|w, a_s) = R = \frac{R_1}{R_0 + R_1} \text{ for all } s, j$$

4. Experiments on synthetic datasets

Figures S1, S2 and S3 show recall, precision and accuracy for the various synthetic datasets, assuming unknown annotations, known annotations, and random annotations, while varying the fraction of pathways presumed active.

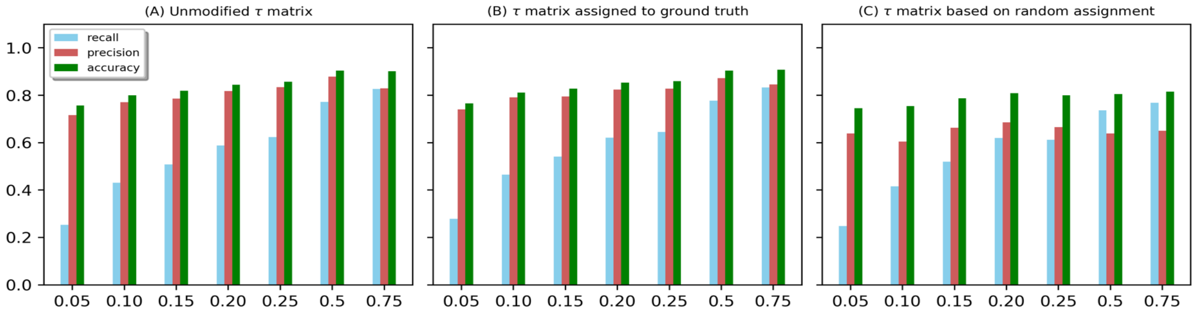

Figure S1. PUMA performance in overcoming uncertainty of multiple assignments. Average recall, precision and accuracy for different experiments in synthetic dataset assuming 0.3 of pathways are active. The x-axis corresponds to the fraction of active metabolites, and y-axis shows correctly identified pathways with multiple candidates. (A) original τ matrix, mapping metabolites to the corresponding mass bin, (B) τ matrix is modified based on ground truth, and (C) τ matrix is modified based on random selection of metabolites.

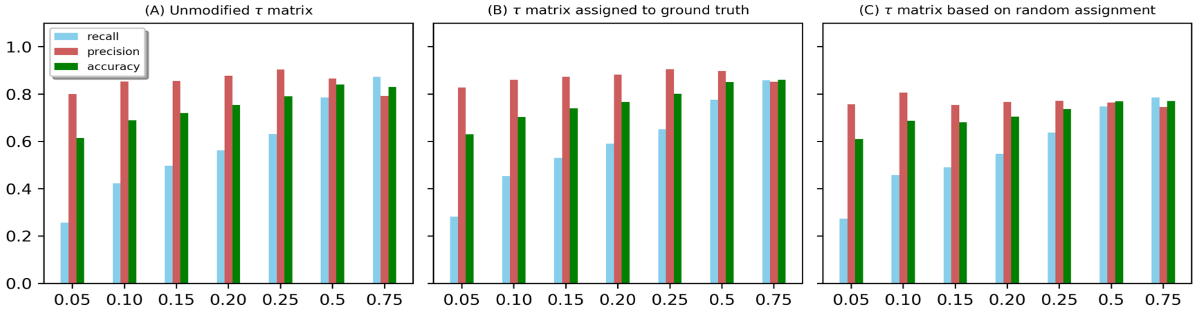

Figure S2. PUMA performance in overcoming uncertainty of multiple assignments. Average recall, precision and accuracy for different experiments in synthetic dataset assuming 0.5 of pathways are active. The x-axis corresponds to the fraction of active metabolites, and y-axis shows correctly identified pathways with multiple candidates. (A) original τ matrix, (B) τ matrix is modified based on ground truth, and (C) τ matrix is modified based on random selection of metabolites.

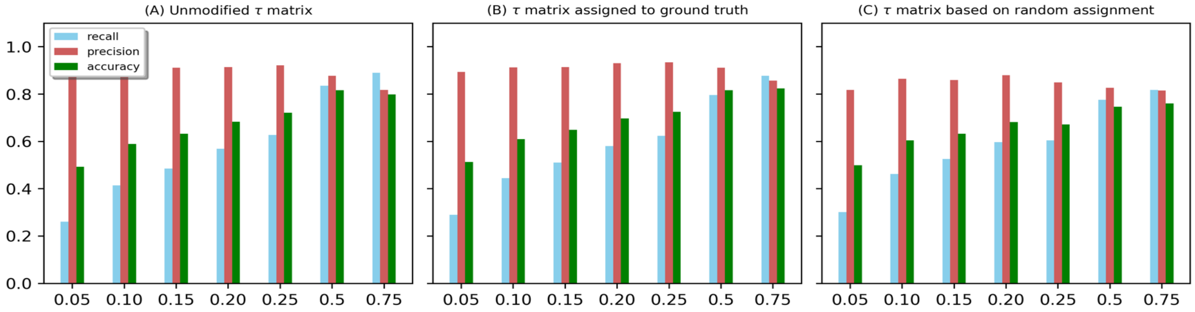

Figure S3. PUMA performance in overcoming uncertainty of multiple assignments. Average recall, precision and accuracy for different experiments in synthetic dataset assuming 0.7 of pathways are active. The x-axis corresponds to the fraction of active metabolites, and y-axis shows correctly identified pathways with multiple candidates. (A) original τ matrix, (B) τ matrix is modified based on ground truth, and (C) τ matrix is modified based on random selection of metabolites.

Figure S4 explores the robustness of the model to parameter μ. For various values of μ, recall, precision, and accuracy seem robust to such variations, indicating that the model is robust to the μ parameter.

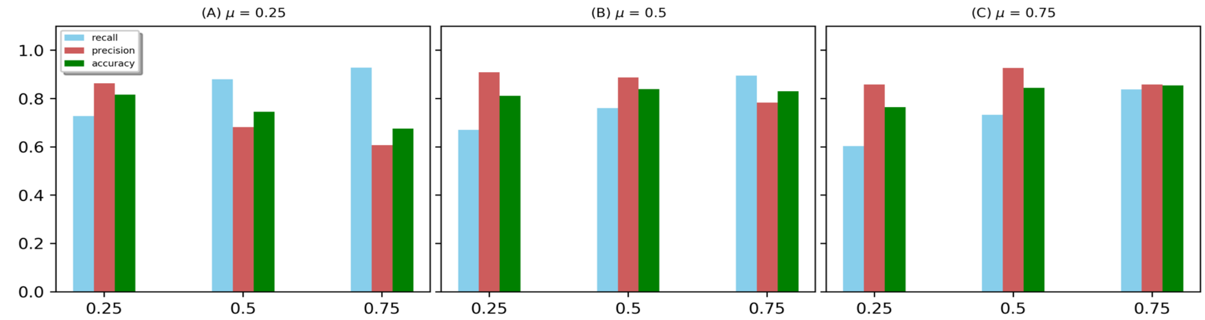

Figure S4. PUMA performance for various values for μ for 0.5 pathway activity with 0.25, 0.50 and 0.75 generated metabolites (x-axis). (A) μ = 0.25. (B) μ = 0.5. (C) μ = 0.75.

5. Additional information for CHO case study

As mass observations differ from one set of measurements to another, the predicted activity differs among the individual CHO cell datasets collected using different instrument settings (dataset HilNeg, HilPos, SynNeg). There are several cases to consider. In some cases, *e.g.* galactose metabolism, purine metabolism, pyrimidine metabolism, tyrosine metabolism, tryptophan metabolism, amino sugar and nucleotide sugar metabolism, folate biosynthesis and metabolism of xenobiotics by cytochrome P450, pathways are predicted active by each individual dataset and the combined dataset. In other cases, *e.g.* selenocompound metabolism and alpha-Linolenic acid metabolism, pathways are predicted active in the combined dataset, but not predicted active for all other individual datasets. In such cases, individual dataset measurements when considered independently of others did not provide inference sufficient evidence to conclude that the pathway is active. As an example, for pathway alpha-Linolenic acid metabolism, with size twelve, the number of mass measurements in SynNeg and combined datasets that can be mapped to the pathway is one. PUMA predicts this pathway active in both datasets. However, the same pathway is not predicted active in HilNeg and HilPos, where the number of mass measurements that can be mapped to the pathway is reduced to zero.

In other cases, some pathways (e.g. glycine, serine and threonine metabolism, beta-alanine metabolism, beta-alanine metabolism and ether lipid metabolism) are predicted active by at least one of the individual datasets while predicted not active by

the combined dataset. Additional evidence in the form of a larger number of mass measurements in the combined dataset affects the predicted activity for pathways with common metabolites. For example, pathway ether lipid metabolism, with size eight, is predicted active by HilNeg but not predicted active by the combined dataset. In both datasets, three mass measurements can be mapped to the pathway, while two of these mass measurements can also be mapped to glycerophospholipid metabolism. With an increase in the number of mass measurements that can be mapped to glycerophospholipid metabolism from 2 in HilNeg to 8 using the combined dataset, glycerophospholipid metabolism has a higher probability of being active compared to ether lipid metabolism. The combined dataset, with the highest number of mass measurements, is the most reliable predictor of pathway activity.
